# Positive involuntary autobiographical memories: You first have to live them

**DOI:** 10.1016/j.concog.2013.01.008

**Published:** 2013-06

**Authors:** Ian A. Clark, Clare E. Mackay, Emily A. Holmes

**Affiliations:** a Department of Psychiatry, University of Oxford, United Kingdom; b Medical Research Council Cognition and Brain Science Unit, Cambridge, United Kingdom

**Keywords:** Involuntary memory, Autobiographical memory, Mental imagery, Emotions

## Abstract

Involuntary autobiographical memories (IAMs) are typically discussed in the context of negative memories such as trauma ‘flashbacks’. However, IAMs occur frequently in everyday life and are predominantly *positive*. In spite of this, surprisingly little is known about how such positive IAMs arise. The trauma film paradigm is often used to generate negative IAMs. Recently an equivalent positive film was developed inducing positive IAMs (Davies, Malik, Pictet, Blackwell, & Holmes, 2012). The current study is the first to investigate which variables (emotional reaction to the film; recognition memory of the film; participant characteristics) would best predict the frequency of positive IAMs. Higher levels of positive mood change to the film were significantly associated with the number of positive IAMs recorded in the subsequent week. Results demonstrate the importance of positive emotional reaction at the time of an event for subsequent positive IAMs.

## Highlights

► First study to investigate the formation of *positive* involuntary autobiographical memories from positively rated films. ► Positive involuntary memories occur frequently and universally. ► Positive mood change at the time of encoding associated with frequency of positive involuntary memories.

## 1 Introduction

Autobiographical memory is the area of memory related to the recollection of past personal events (Conway & Pleydell-Pearce, 2000). In some cases autobiographical memory occurs via a voluntary or deliberate process – actively recalling past events to remember a particular detail or to relive an experience. In contrast, involuntary memories are those which are spontaneously brought to consciousness without preceding attempts to retrieve them (Berntsen, 1996; Mace, 2007). A recent study using a mechanical counter found that involuntary autobiographical memories (IAMs) occurred three times as frequently as voluntary memories over the course of a normal day in everyday life (Rasmussen & Berntsen, 2011). Additionally, a telephone survey of 1500 Danes identified that approximately 60% of IAMs reported were positive in nature (Berntsen & Rubin, 2008). Such IAMs may help us spontaneously relive our past positive experiences in the texture of everyday life and can improve our mood in the process.

We know surprisingly little about how or why such positive IAMs arise, although more is known about negative IAMs. Negative IAMs have been well researched in the form of ‘flashbacks’; a common and distressing symptom of Posttraumatic Stress Disorder (PTSD; American Psychiatric Association, 2000). Most commonly, flashbacks occur as image based negative involuntary autobiographical memories of traumatic events which hijack attention (Clark, Holmes, & Mackay, in press). Hence, in this form they are similar to that of positive IAMs, differing only in their emotional valence.

To our knowledge, there has only been one previous study examining involuntary memories of potentially positive rather than negative film material. This study from 1973 compared stressful films with ‘erotic’ films and studied participants ‘mental content’ while performing a signal detection task (Horowitz & Becker, 1973). They reported no difference in the frequency of intrusive mental content (partially related to IAMs) to the two films, and this frequency was inversely correlated with negative, but not positive, emotional reactions to the erotic film. Thus, it is not clear whether the intrusive mental content of the erotic film was emotionally positive for participants. It would therefore be of interest to test a film with overtly positive material and to measure positive IAMs directly.

Research into flashback development may be able to inform our understanding of positive IAMs. Emerging research suggests that flashback processes are similar to general memory formation (Berntsen & Rubin, 2008; Krans, Näring, & Becker, 2009). An individual’s emotional response during and immediately after a traumatic event is one of the strongest predictors of PTSD (Ozer, Best, Lipsey, & Weiss, 2003). Individual characteristics, (e.g. gender, history of depression, anxiety) have also been found to predict PTSD but to a lesser extent (Brewin, Andrews, & Valentine, 2000; Ozer et al., 2003). Additionally, patients with bipolar disorder frequently report flashback memories (Gregory, Brewin, Mansell, & Donaldson, 2010). Emotional reaction during an event and some participant characteristics may therefore also be important for the formation of positive IAMs.

A commonly used methodology to investigate flashback development is the trauma film paradigm (Holmes & Bourne, 2008). The trauma film paradigm involves participants watching traumatic film footage (e.g. car crashes and surgery) as an analogue of real trauma in the laboratory (Bourne, Mackay, & Holmes, in press). Participants then keep a diary to monitor any subsequent IAMs of the film footage over the following week (Holmes, Brewin, & Hennessy, 2004) – a widely used methodology in IAM literature (e.g. Berntsen, 2001; Laposa & Alden, 2008). Additionally, Schlagman and Kvavilashvili (2008) found no differences between IAMs reported in a written diary and IAMs reported during an undemanding vigilance task conducted in the laboratory. As a method of self-report, the written diary could be argued to have distorting effects, however, the diary method offers a unique window to understand phenomenology, in this case IAMs, in the context of everyday life (see Bolger, Davis, & Rafaeli, 2003 for a detailed review on the strengths and limitations of the diary methodology).

Recently, an equivalent ‘positive’ film paradigm was developed successfully eliciting positive IAMs (Davies et al., 2012). Here we used a positive film, including, for example, scenes that encompassed the jubilation of being enthusiastically greeted after finishing university final exams, the excitement of a rollercoaster ride and extreme sports, the pride of graduating, and the thrill of gambling. The current experiment investigated the influence of state and trait variables on the frequency of positive IAMs, predicting that film viewing variables (emotional reaction to the film, recognition memory of the film), would have a greater effect than participant characteristics (age, gender, history of hypomania, depression, anxiety).

## 2 Method

### 2.1 Participants and procedure

**Fig. 1:**
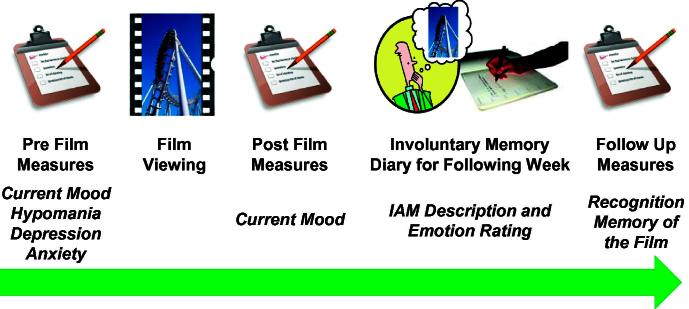
Diagram showing the experimental procedure. Participants filled out questionnaires concerning their current mood and baseline characteristics before watching the positive film. After film viewing mood was reassessed and participants were asked to record any involuntary memories of the film over the following week. Participants returned a week later and completed a recognition memory test of the film.

The sample consisted of 95 participants (53 female) with a mean age of 23.45 years (*SD *= 7.0). On arrival, participants were asked to complete questionnaires concerning their current mood and baseline characteristics (Section 2.2). Participants were then asked to watch the positive film, imagining that the events being depicted were happening to them right now. After film viewing, participants’ mood was reassessed and they were asked to record any IAMs of the film in a 1-week diary (Holmes et al., 2004). Participants returned after one week and completed a recognition memory test of the film (Fig. 1).

### 2.2 Measures

#### 2.2.1 Participant characteristics

History of hypomania was measured using the Mood Disorders Questionnaire (MDQ; Hirschfield et al., 2000). The self-report questionnaire is split into three sections; Section 1 consists of 13 yes/no items looking at lifetime (hypo)manic symptoms, Section 2 asks about symptom co-occurrence, and Section 3 asks about symptom severity on a four point scale (no problem, minor problem, moderate problem, serious problem). Total scores range from 0 to 17, with severity scored from 0 to 3, respectively. Higher scores represent higher levels of hypomanic history.

Current depression levels were measured using the Beck Depression Inventory Second Edition (BDI-II; Beck, Steer, & Brown, 1996). Participants respond to 21 questions on a scale of 0 to 3, asking about their mood over the last 2 weeks. Higher total scores represent higher levels of current depression.

Trait anxiety was measured using the State-Trait Anxiety Inventory-Trait version (STAI-T; Spielberger, Gorsuch, Lushene, Vagg, & Jacobs, 1983). The STAI-T contains 20 anxiety related items which participants’ rate on a four point scale as to how they generally feel. Higher total scores represent higher levels of trait anxiety.

#### 2.2.2 Film viewing variables

Emotional reaction to the film was assessed by the positive subscale of the Positive and Negative Affect Schedule 10 (PANAS-10; Watson, Clark, & Tellegen, 1988) before and after film viewing. Scores on the positive subscale can range from 10 to 50, with higher scores representing higher positive affect. Emotional reaction to the film (residual PANAS mood change) was determined from the standardised residuals of the PANAS-10 change scores to take into account mood before the film (as in Laposa & Alden, 2008).

Recognition memory of the film was assessed one week after film viewing using a 38-item forced choice visual recognition memory test. The recognition memory test comprised 19 stills from the film and 19 stills from unused sections of clips that had been edited out of the film or were from similar situations (Wegner, Quillian, & Houston, 1996).

#### 2.2.3 Involuntary memory diary

IAMs of the film were recorded in an involuntary memory diary (Holmes et al., 2004). For each IAM, participants wrote a brief description and rated the emotion on a 5-point scale from *very negative* to *very positive* (Berntsen, 2001). A rating of 4 or 5 was classed as a positive IAM and 3 as a neutral IAM.

## 3 Results

In the involuntary memory diaries, 186 positive IAMs of the film were reported (mean = 1.96, *SD* = 2.34; range 0–11), with 65% of participants reporting at least one positive IAM. IAMs could be of the same or different scenes per participant; the mean number of different scenes was 1.39 (*SD* = 1.48).

Prediction of positive IAM frequency was assessed using separate regression analyses for film viewing variables and participant characteristics. Depression scores were skewed and cube-rooted (BDI-IIcr). As IAMs are count data, negative binomial regression was used to fit the models (Gardner, Mulvey, & Shaw, 1995).

The regression models fitted the data well [Pearson statistic for general linear models: film viewing: *χ*^2^ = 85.33 (*df = *92, *p* = .40); participant characteristics: *χ*^2^ = 83.70 (*df* = 89, *p* = .14)]. Within the film viewing variables, residual PANAS mood change (i.e. the change in score on the positive subscale, with participants mood before film viewing taken into account) significantly predicted the frequency of positive IAMs. That is, all else being equal, the odds of a positive IAM occurring increased by 1.6 for each 1 point increase in residual PANAS mood change. Thus, as positive mood change increases, so does the likelihood of a positive IAM (Table 1).

Neither voluntary memory nor any participant characteristic was found to significantly predict positive IAMs (Table 1).

To better understand the relationship between positive mood change and positive IAMs, we investigated whether emotional reaction to the film was also associated with neutral IAM frequency. In addition to the positive IAMs, participants reported 148 neutral IAMs (mean = 1.56, *SD* = 1.75; range 0–8). A weak but significant correlation was found between positive and neutral IAM frequency (*r* = 0.25, *p* = .015). Prediction of neutral IAM frequency was also assessed using a negative binomial regression model. As the only significant predictor of positive IAMs residual PANAS mood change was the only predictor entered into the model. The regression model fitted the data well (*χ*^2^ = 73.23, *df* = 93, *p* = .62). Residual PANAS mood change significantly predicted the frequency of neutral IAMS; all else being equal, the odds of a positive IAM increased by 1.32 for each 1 point increase in residual PANAS mood change (Table 1).

## 4 Discussion

To the best of our knowledge, this is the first study to investigate in detail the formation of positive IAMs from overtly positive stimuli. The results highlight the importance of emotional reaction at the time of a positive event for the formation of positive and neutral IAMs. Interestingly, the extent of the increase in IAM frequency seems to be valence dependent – the odds ratio predicting the occurrence of positive IAMs was higher than for neutral IAMs. Additionally, the results support the notion that IAMs are not unique to negative and distressing events in the form of flashbacks, also occurring for positive information (Berntsen, 2001; Berntsen & Rubin, 2008; Krans et al., 2009).

The current study found no relationship between positive IAMs (involuntary memories) and recognition memory for the film. That is, these types of memory may behave independently. Recent work suggests that involuntary and voluntary autobiographical memories share the same episodic memory encoding systems, but differ in their retrieval (Berntsen, 2010). The current study is consistent with this view point. In the current study, positive emotional reaction at the time of film viewing predicted both positive and neutral IAMs. Related to this – emotional intensity at the time of encoding has been found to be important for the successful recall of voluntary autobiographical memories (Talarico, LaBar, & Rubin, 2004).

None of the participant characteristics we included were associated with positive IAMs. The lack of an association between history of hypomania and positive IAMs from the film may be for several reasons. Bipolar patients experience involuntary images during periods of elevated mood, but these are often of future events rather than past events (Gregory et al., 2010; Ivins, Di Simplicio, Close, Goodwin, & Holmes, submitted for publication). There may therefore be important differences in the formation of involuntary future images and IAMs. However, fundamentally, it is still a question whether ‘positive’ is a genuine link to hypomania; positive biases in cognition do not predict bipolar disorder, while negative biases do (Kelly et al., 2011). History of hypomania may therefore not be enough to predict positive IAMs. In terms of depression and anxiety, the non significant finding may be due to high levels of depression and anxiety being associated with a bias for negative and fearful stimuli (Mathews & MacLeod, 2005). This bias enhances processing of negative events, for example trauma, but may not also enhance processing for positive events.

The current results may also have relevance for the treatment of emotional disorders. Positive autobiographical memories are lacking in depression (Werner-Seidler & Moulds, 2011) and understanding how positive IAMs are formed may have implications for increasing positive memories. On the other hand, positive involuntary images may be involved in the maintenance of manic episodes in bipolar disorder (Holmes, Geddes, Colom, & Goodwin, 2008; McCarthy-Jones, Knowles, & Rowse, 2012). Understanding the mechanisms of IAMs may lead to the development of treatments that aim to manipulate the frequency of IAMs relevant to the specific nature of the disorder.

Future work investigating positive IAMs should include a free recall test to explore whether there is a difference between various measures of involuntary and voluntary autobiographical memory retrieval. Additionally, it would be interesting to include convergent measures to the diary of IAMs to address possible limitations of the diary methodology.

In conclusion, the current study found that positive emotional reaction at the time of experiencing a positive event best predicted the frequency of later positive IAMs. Thus, in tune with a quote attributed to Bob Dylan, “If you want to keep your memories, you first have to live them”.

## Figures and Tables

**Table 1:** Negative binomial regression models showing that residual PANAS mood change over the film significantly predicted the frequency of positive and neutral IAMs over 1 week.

Variable	*B*	*SE*	OR	95% CI
*Positive IAMs film viewing model*				
Intercept	0.70	1.09	2.01	[0.24, 16.93]
Residual PANAS mood change	0.47	0.13	1.60⁎⁎⁎	[1.23, 2.06]
Recognition memory of the film	−0.0015	0.014	1.00	[0.97, 1.03]

*Positive IAMs participant characteristics model*				
Intercept	0.71	0.73	2.03	[0.48, 8.52]
Age	0.0039	0.018	1.00	[0.97, 1.04]
Gender [Male coded as 1]	0.14	0.25	1.15	[0.70, 1.90]
MDQ	0.023	0.031	1.02	[0.96, 1.09]
BDI-IIcr	−0.22	0.20	0.81	[0.55, 1.19]
STAI-T	−0.00066	0.013	1.00	[0.97, 1.03]

*Neutral IAMs mood change model*				
Intercept	0.41	0.12	1.51⁎⁎⁎	[1.20, 1.91]
Residual PANAS mood change	0.28	0.12	1.32⁎	[1.04, 1.67]

*Note:* OR = Odds Ratios; CI = Confidence Intervals; MDQ = Mood Disorders Questionnaire; STAI-T = State Trait Anxiety Inventory Trait version; BDI-IIcr = Beck Depression Inventory Second Edition, cube rooted. ^∗∗^*p* < 0.01.

⁎⁎⁎ *p* < 0.001.

⁎ *p* < 0.05.
